# Supplementary material for: Neural network enabled nanoplasmonic hydrogen sensors with 100 ppm limit of detection in humid air
Source: Nat Commun. 2024 Feb 8;15:1208. doi: 10.1038/s41467-024-45484-9 (PMC10853499; doi:10.1038/s41467-024-45484-9)
Supplement: Supplementary file 1 — Supplementary Information [file 41467_2024_45484_MOESM1_ESM.pdf]

# Supplementary Information for

# Neural network enabled nanoplasmonic hydrogen sensors with 100 ppm limit of detection in humid air

*David Tomeček[1], Henrik Klein Moberg[1], Sara Nilsson[1], Athanasios Theodoridis[1], Iwan Darmadi[1], Daniel Midtvedt[2], Giovanni Volpe[2], Olof Andersson[3] and Christoph Langhammer[1,]**

[1]Department of Physics, Chalmers University of Technology, 412 96 Göteborg, Sweden

[2]Department of Physics, University of Gothenburg, 412 96 Göteborg, Sweden

[3]Insplorion AB, Arvid Wallgrens Backe 20, 413 46, Göteborg, Sweden

*clangham@chalmers.se

**Supplementary Section 1: Sieverts' law dependency in dry conditions at 30°C**

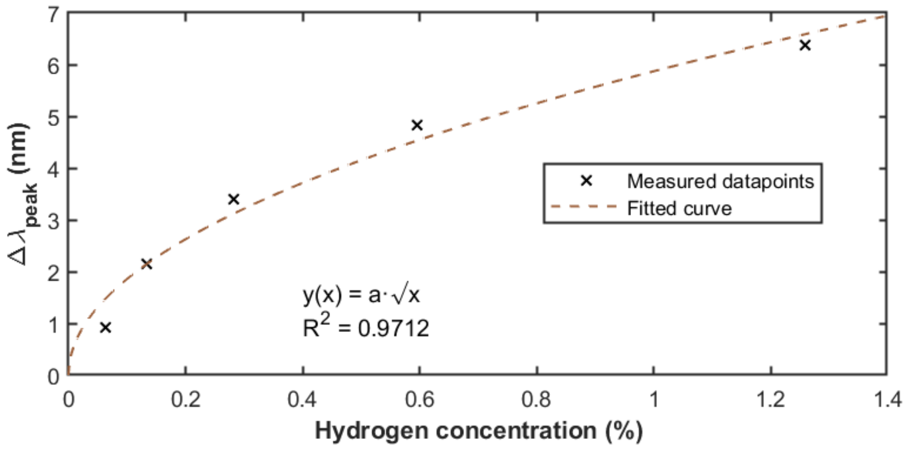


***Supplementary Figure 1.*** *Sieverts' law fit to the $\Delta\lambda_{peak}$ – $c_{H2}$ dependence measured at dry conditions.*

**Supplementary Section 2: Sensor baseline spectral peak position, $\lambda_{peak}$, as a function of ~~a~~ humidity level at 30 °C**

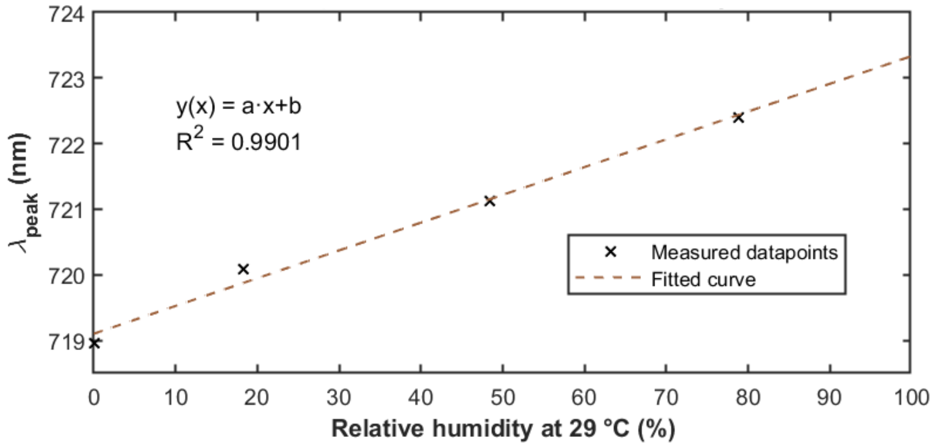


***Supplementary Figure 2.*** *Impact of humidity on the sensor baseline peak position, $\lambda_{peak}$, at 30 °C. Clearly, the sensor baseline elevates linearly with relative humidity (RH).*

**Supplementary Section 3: Comparison of initial and closing sets of sensor response at 30°C in dry conditions**

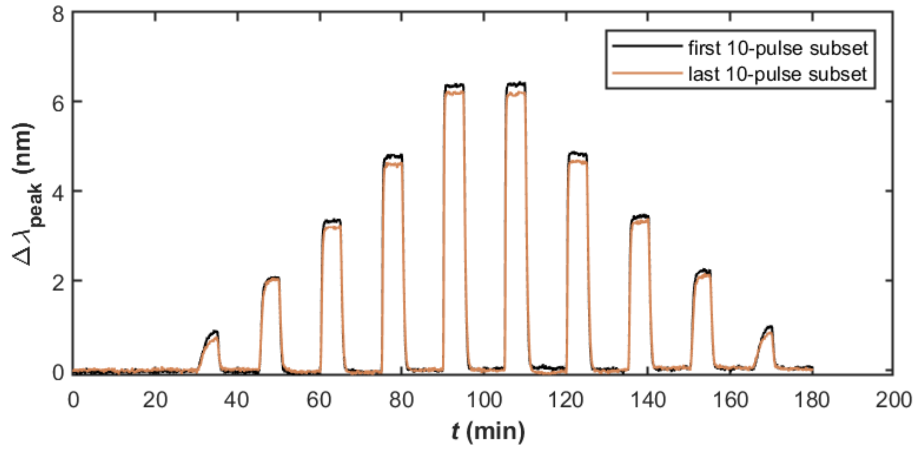


***Supplementary Figure 3.*** *Comparison of the initial and the closing set of sensor response obtained in dry conditions as extracted from Figure 2 in the main text.*

**Supplementary Section 4: $\Delta\lambda_{peak}$ vs. $H_2$ concentration in all humidities at 30 °C**

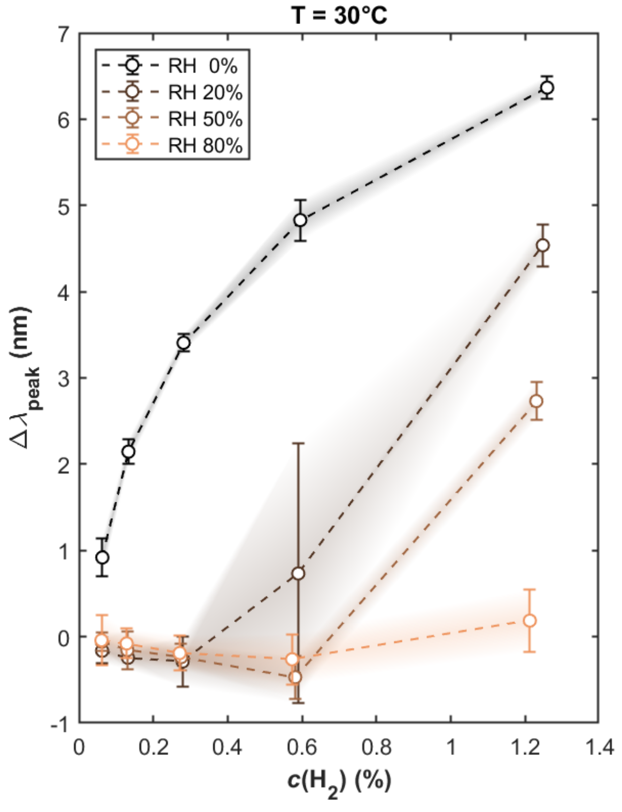


***Supplementary Figure 4.*** *$\Delta\lambda_{peak}$ as a function of $H_2$ concentration, $c_{H2}$, for different RH values at 30 °C, revealing distinct deviation from Sieverts' law for RH > 0. Error bars correspond to three times the standard data deviation, 3σ, containing both repetition and signal noise components. This figure corresponds to Figure 2e in the main text and is reproduced here in larger format for clarity.*

**Supplementary Section 5: Fundamental difference between dry and humid conditions**

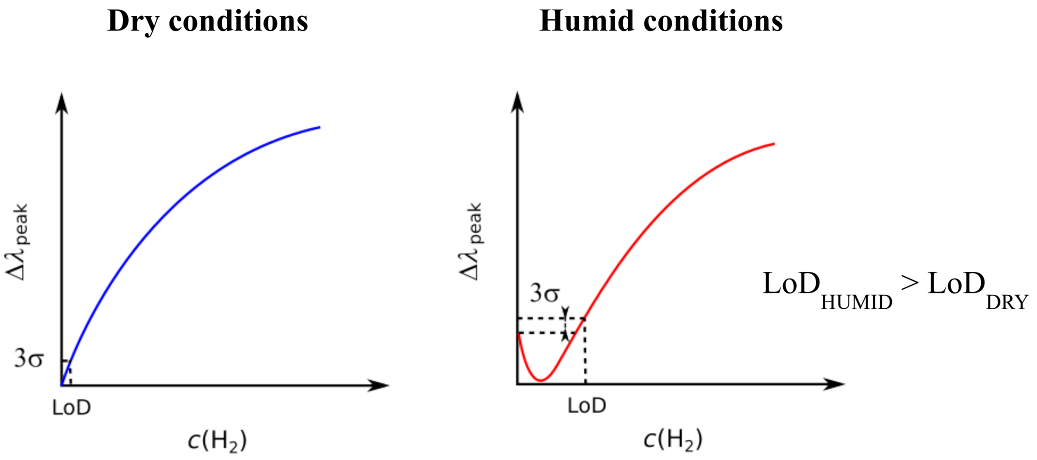


***Supplementary Figure 5.*** *Schematic illustration of the relations between $\Delta\lambda_{peak}$ and $c_{H2}$ in dry (blue) and humid (red) conditions with the blue-shift (negative response) region at low $H_2$ concentrations in humid conditions as the key feature.*

**Supplementary Section 6: Structure of the algorithm used to calculate the LoD of the $\Delta\lambda_{peak}$ readout**

1. Data noise calculation ($\sigma$) – 60 datapoints (3 min) taken before the 1st exposure to $H_2$ at each temperature and relative humidity level

2. Five concentrations measured. Finding the first concentration where the response > 3σ

3. Branching: if it is fulfilled by the first concentration point [$c_1$, $\Delta\lambda_{peak,1}$] --> branch A, otherwise branch B

4A. Linear interpolation between [0,0] point and [$c_1$, $\Delta\lambda_{peak,1}$] point to find the concentration where the response would be equal to $3\sigma$

(semi-logarithmic extrapolation* from [$c_1$, $\Delta\lambda_{peak,1}$] and [$c_2$, $\Delta\lambda_{peak,2}$] was also tested and provides similar results)

4B. Semi-logarithmic interpolation* between [$c_{n-1}$, $\Delta\lambda_{peak,n-1}$] and [$c_n$, $\Delta\lambda_{peak,n}$] where $\Delta\lambda_{peak,n}$ is the first response greater than 3σ to find the concentration where the response would be equal to 3σ

(linear interpolation between [$c_{n-1}$, $\Delta\lambda_{peak,n-1}$] and [$c_n$, $\Delta\lambda_{peak,n}$] provides comparable values)

*linear extrapolation/interpolation in semi-logarithmic coordinates (with $\Delta\lambda_{peak}$ linear and $c_{H2}$ logarithmic)

## Supplementary Section 7: Sensor response at 55 °C, RH = 80%

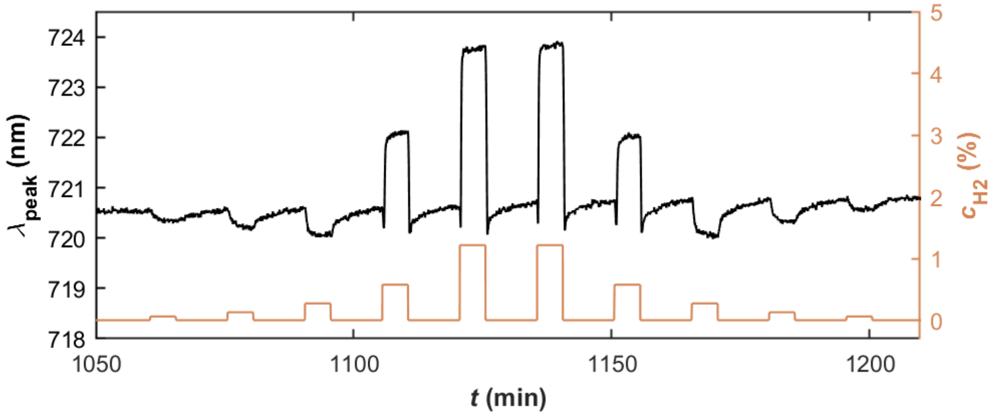


***Supplementary Figure 6.*** *$\lambda_{peak}$ recorded at 55 °C and RH = 80%. Zoomed data from Figures 3a,b in the main text.*

## Supplementary Section 8: Definition of the LoD for a sensor operating at 55 °C at RH 80% using $\Delta\lambda_{peak}$ readout

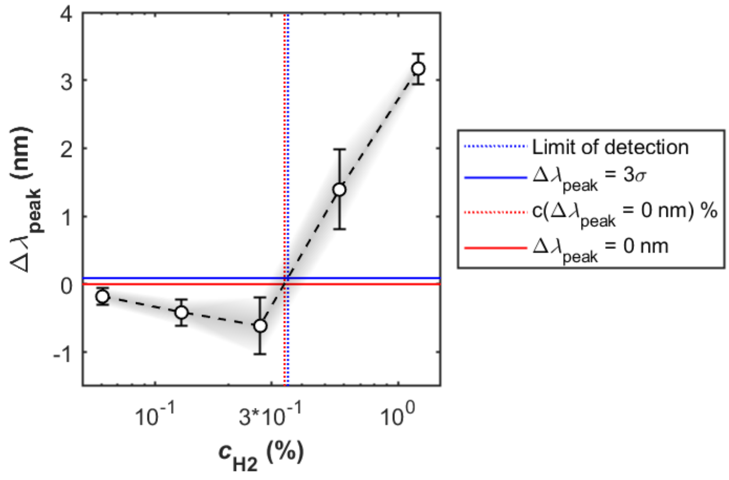


***Supplementary Figure 7.*** *$\Delta\lambda_{peak}$ as a function of $c_{H2}$ at 55 °C and RH = 80%. Blue lines illustrate the estimated LoD defined as the first distinguishable positive $\Delta\lambda_{peak}$. Red lines illustrate the estimate of the positive $\Delta\lambda_{peak}$ threshold, i.e., $c_{H2}$ at which $\Delta\lambda_{peak}$= 0 nm.Error bars correspond to three times the standard data deviation, 3σ, containing both repetition and signal noise components – details in* ***section 17.***

**Supplementary Section 9: Positive $\Delta\lambda_{peak}$ response threshold as a function of RH and temperature T**

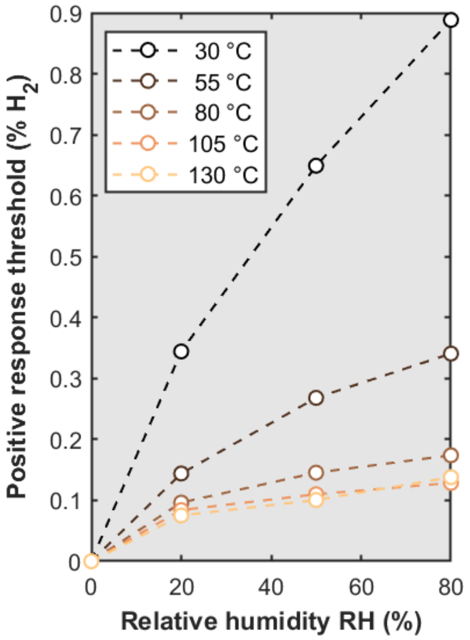


***Supplementary Figure 8.*** *Positive $\Delta\lambda_{peak}$ response threshold as a function of relative humidity (RH) and sensor operating temperature. The positive response threshold corresponds to a non-zero $c_{H2}$ for which the sensor returns $\Delta\lambda_{peak}$ = 0 nm before returning positive $\Delta\lambda_{peak}$ values for further increasing $c_{H2}$ values.*

**Supplementary Section 10: Alternative visualization of the data in Figure 3c in main text**

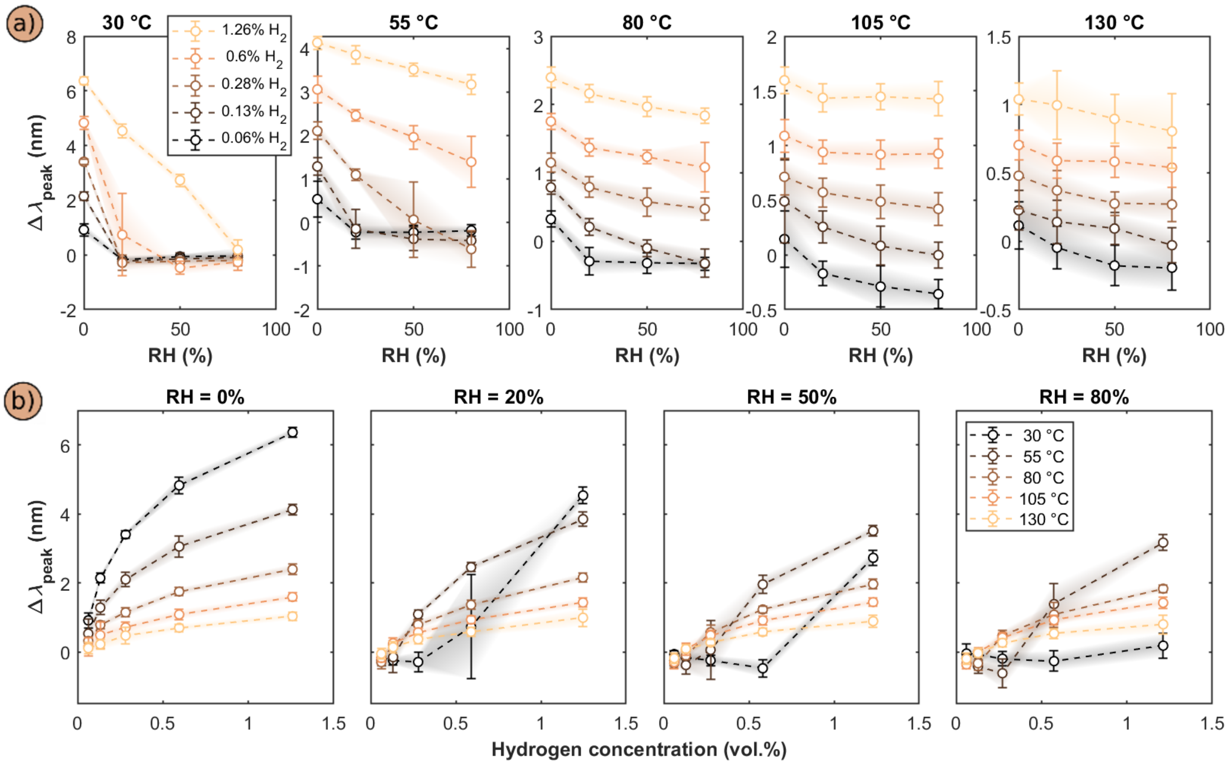


***Supplementary Figure 9.*** *Temperature effects on hydrogen sensing performance in humid environment – alternative visualizations of the data depicted in Figure 3c in the main text: a) $\Delta\lambda_{peak}$ as a function of relative humidity for various hydrogen concentrations and temperatures; b) $\Delta\lambda_{peak}$ as a function hydrogen concentration for various temperatures and relative humidities. Error bars correspond to three times the standard data deviation, 3σ, containing both repetition and signal noise components – details in* ***section 17.***

**Supplementary Section 11: $\Delta\lambda_{peak}$ sensor signal noise**

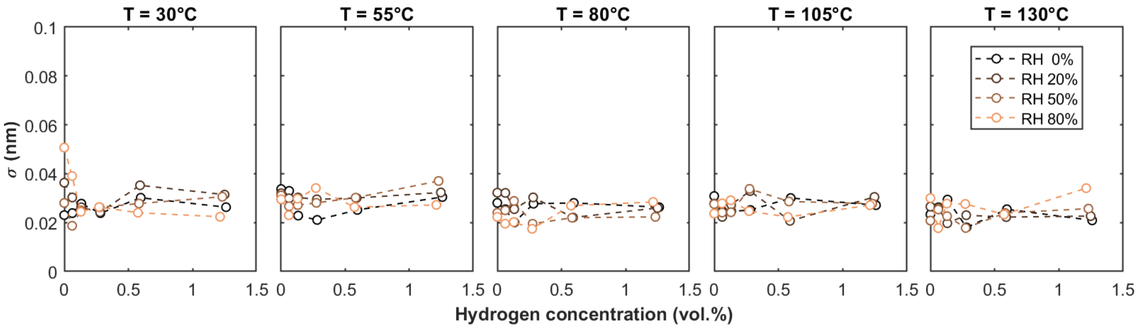


***Supplementary Figure 10.*** *Noise level, expressed as standard deviation in the $\Delta\lambda_{peak}$ signal, plotted as a function of hydrogen concentration, relative humidity, and sensor operating temperature.*

**Supplementary Section 12: Deep Dense Neural Network (DDNN) architecture**

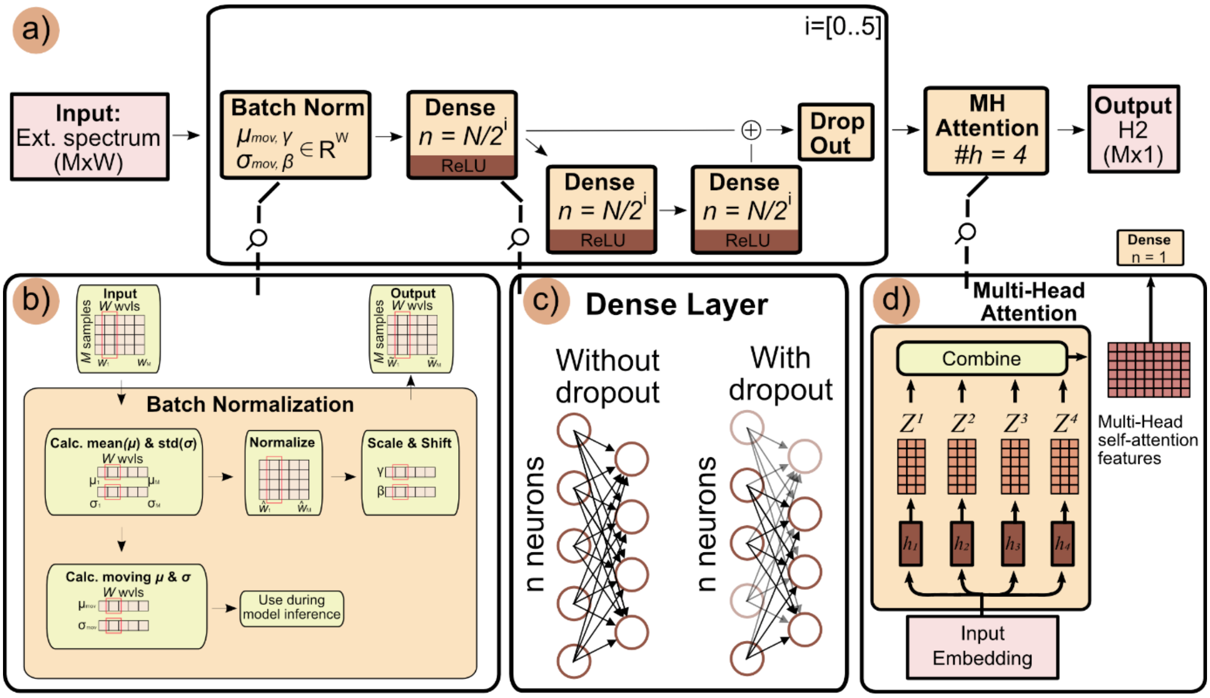


***Supplementary Figure 11.*** *Illustration of the DDNN-based architecture.*

Neural networks are effectively piecewise linear function approximators, capable in principle of approximating any function through finding hyperplane partitions of the input data. The depth of a neural network affects how complex these partitions might grow, in essence improving the representational ability of the whole architecture. Effectively, subsequent layers of neural networks (assuming equal or growing layer size) increase the dimensionality of the input data to the extent where finding some hyperplanar partition to approximate the output values is always possible. This forms the basis of the dense neural network architecture used in this study, where we employ an architecture to map directly between extinction spectrum and hydrogen concentration separately in each timestep. To prevent biasing the results and ensure that all information in the spectrum (input) is equally represented, we employ a fully connected dense neural network with each individual neuron in the input layer connected to each extinction value of an input spectrum and thereby incorporate full spectral information for each mapping. In this configuration, using a direct 1:1 mapping between input and output, the numerical correlations will be highly sensitive to drift and spurious measurement noise thereby necessitating a robust normalization scheme. To accomplish this, apart from a min-max normalization using an initial calibration curve for each separate measurement series, we include batch normalization BatchNorm[1] (**Supplementary Figure 11b**) layers which learn suitable normalization parameters during training given the training data and freeze them during inference. Finally, to improve the likelihood of the network finding physically relevant correlations which generalize properly across datasets, we introduce Dropout[2] (**Supplementary Figure 11c**) layers which randomly reset network weights during training and thereby force the network to learn strong (i.e. not spurious) correlations robust to random variability. To improve the computational power available to the network and increase its representational ability and flexibility, we connect a sequence of 3 dense layers with a

BatchNorm and Dropout layer through a skip connection and connect this structure with 6 identical structures (**Supplementary Figure 11a**). The skip connections are used to mitigate the problems of vanishing gradient with such a deep network.[3] Finally, this feeds through a Multi-head Attention module[4], which performs parallel self-attention computations in $\#h = 4$ heads (**Supplementary Figure 11d**). This allows the model to learn multiple representations of the output of the DDNN sequence in parallel, since each head exploits the underlying relational structure of the data differently. This is similar to, e.g., the concept of filters in convolutional neural networks (CNNs), where different filters learn different representations of an image. The different representations learned by the heads are then combined to produce a single value of hydrogen concentration, which is connected to an L1 norm loss and trained through backpropagation. Everything is implemented with TensorFlow.[5]

### Supplementary Section 13: DDNN architecture training

The network is trained, validated and tested on a bipartition set of the sequential data found in **Figure 3** and **Supplementary Figure 12**, consisting of the sequence of on-ramps of increasing hydrogen concentration and off-ramps of decreasing hydrogen concentration. As illustrated in **Supplementary Figure 12**, the model is trained and validated on 80% and 20% of a random permutation of datapoints in the sequence of on-ramps of increasing hydrogen concentration, respectively. The test data, on which the model is run in inference mode (particularly important for freezing the parameters of BatchNorm layers, see **Supplementary Figure 11**), consists of the orthogonal sequence of off-ramps and is used to derive all results of the DL-based analysis in the main text. A singular model is trained for all combinations of humidity and temperature. The network is trained using holdout validation with a patience of 3000 epochs using the ADAM optimizer[6] with an exponentially decaying learning rate from 1e-5 to 1e-6 with default parameters on a NVIDIA GeForce RTX 3080 GPU. The input during training is a randomly shuffled sequence of spectra and corresponding hydrogen concentration in time with batch size 8192, wherein each spectrum-concentration pair is processed separately

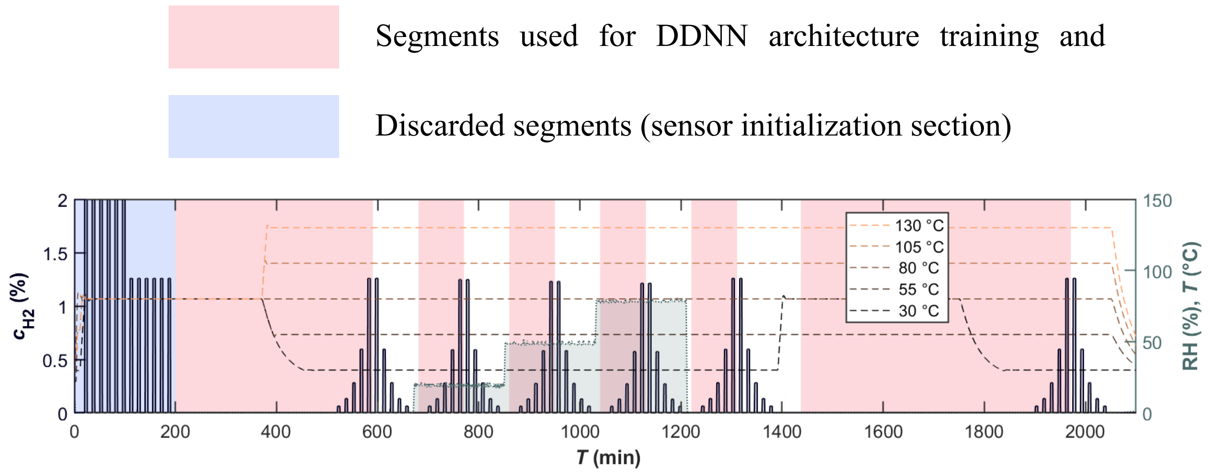


***Supplementary Figure 12.*** *Illustration of dataset segmentation for the purposes of DDNN architecture learning, validation, and testing. The light-red shaded segments were used for DDNN architecture training (80% randomly selected datapoints) and validation (the remaining 20% datapoints). The non-shaded segments (white background) were used for DDNN architecture testing. The light-blue segment was discarded as it serves for sensor initialization only.*

**Supplementary Section 14: Comparison of $\Delta\lambda_{peak}$ and DDNN architecture-based readouts at 80 °C and all RH**

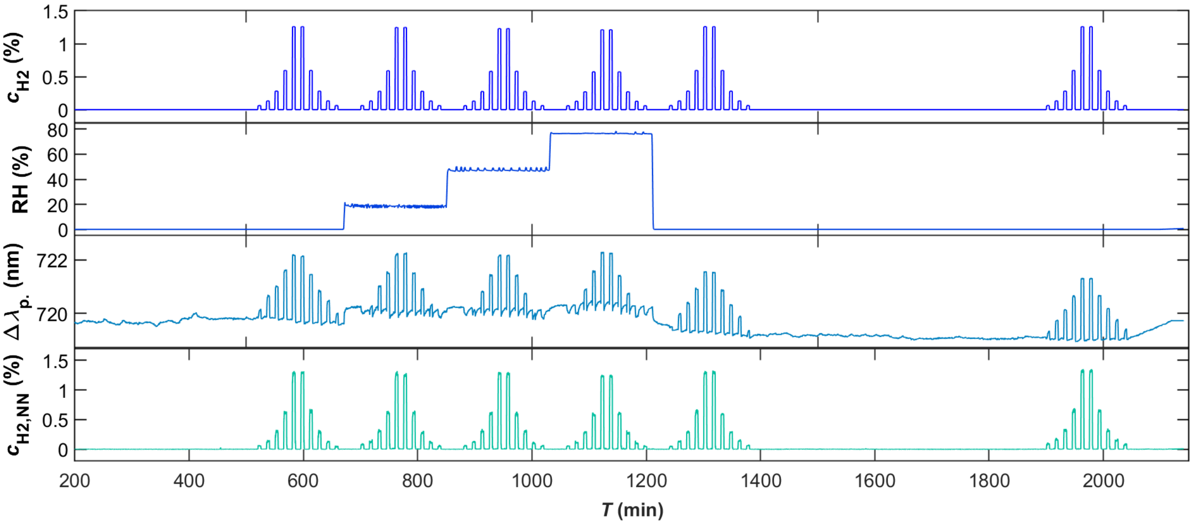


***Supplementary Figure 13.*** *Direct comparison of* $\Delta\lambda_{\text{peak}}$ *(light blue) and DDNN architecture-based (*$c_{\text{H2,NN}}$*, cyan) sensor readouts at 80 °C. The top row in the graph depicts the nominally set* $c_{\text{H2}}$ *values and the second row from the top the set RH.*

**Supplementary Section 15: Comparison of DDNN architecture and $\Delta\lambda_{peak}$ readout across all T and RH**

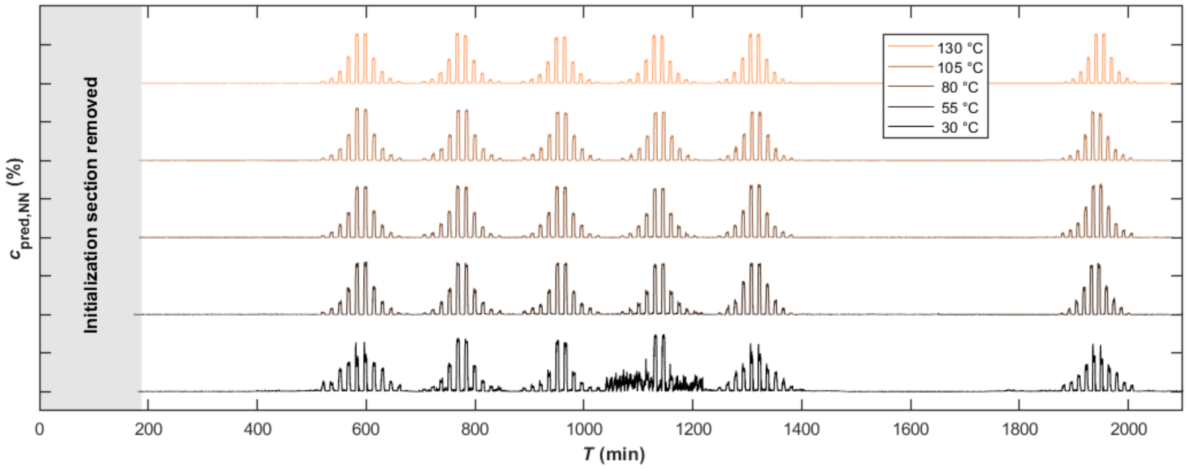


***Supplementary Figure 14.*** *Hydrogen concentration predicted by the DDNN architecture for signals recorded for different humidity levels and sensor operation temperatures. The DDNN architecture uses the same spectral data based on which the* $\Delta\lambda_{peak}$ *values in Figure 3 in the main text (and in* ***Supplementary Figure 15*** *below, for direct comparison) were calculated.*

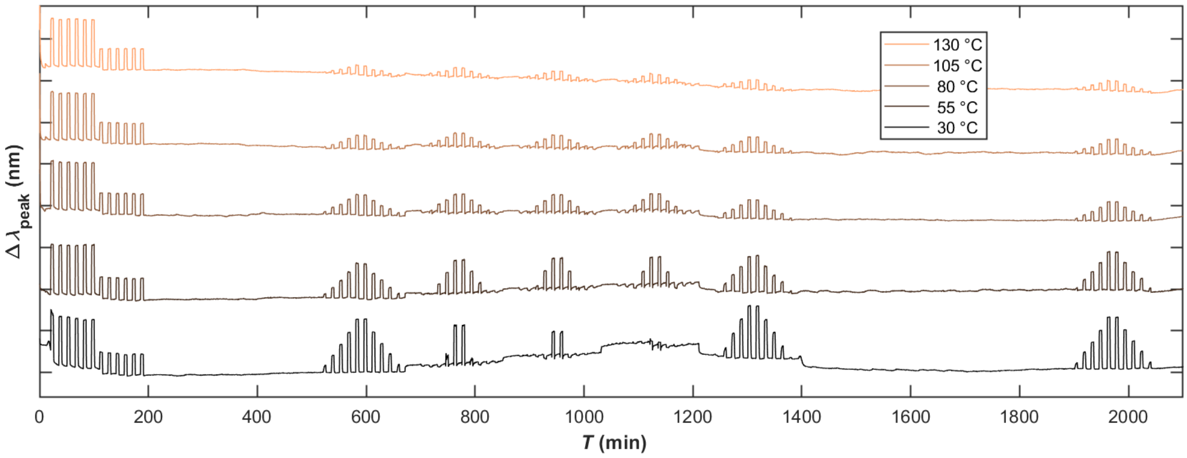


***Supplementary Figure 15.*** $\Delta\lambda_{peak}$ *for various humidity levels and sensor operation temperatures. Identical to Figure 3b in the main text, added here for easy comparison with* ***Supplementary Figure 14****.*

**Supplementary Section 16: LoD calculation for the DDNN architecture data**

The predicted value of each hydrogen concentration $c_{H2}$ is calculated as follows. For each pulse of hydrogen excluded from the training and validation of the DDNN architecture, corresponding to the non-shaded regions of **Supplementary Figure 12**, the DDNN architecture's predicted value of $c_{H2}$ is taken as the mean predicted value of $c_{H2}$ across the whole pulse, excluding the first 50 and last 15 seconds. Similarly, the standard deviation $\sigma$ is obtained through the standard deviation of predicted $c_{H2}$ across each pulse excluding the first 50 and last 15 seconds. Thus, for each separate pulse of $c_{H2}$, we obtain a single predicted value of $c_{H2}$ from the network's prediction across the entire pulse, alongside the corresponding standard deviation.

With this definition, we acquire a single value of $\sigma$ for each discrete value of hydrogen concentration, relative humidity and temperature. To calculate the expected limit of detection from this data, defined as the smallest concentration in which the mean predicted value is still within three standard deviations of prediction, we follow two steps. Firstly, we fit a logarithmic function using scipy[7] to the standard deviation $\sigma$ ($c_{H2}$) as function of hydrogen concentration to obtain a continuous approximation $\sigma$ ($c_{H2\text{-}c}$) of how the network's standard deviation of predictions vary as function of concentration for each separate value of humidity and temperature as shown in **Supplementary Figure 16** for the case of 55°C and 80% RH. Secondly, we calculate the LoD as the smallest $c_{H2}$ where mean prediction $\mu$ ($c_{H2}$ -3$\sigma$ ($c_{H2\text{-}c}$))>0, i.e., the smallest hydrogen concentration which can be predicted with a precision of 3$\sigma$ ($c_{H2}$).

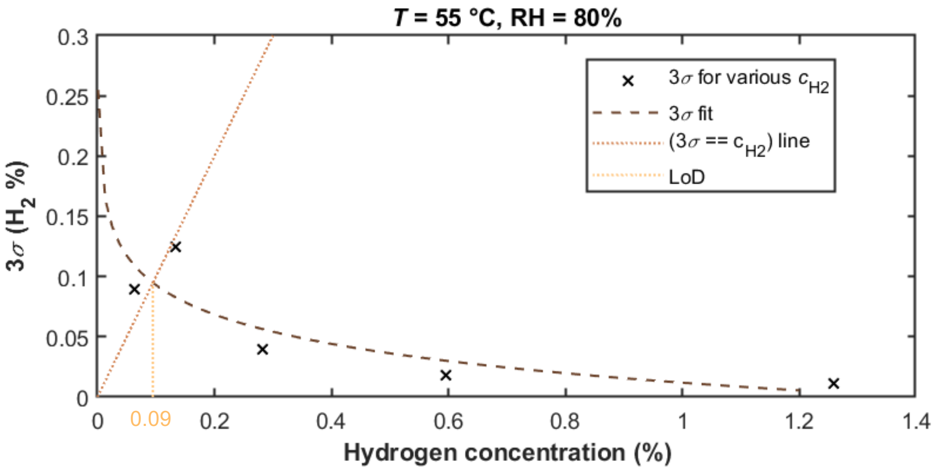


***Supplementary Figure 16.*** *Prediction uncertainty for various hydrogen concentrations at the operating temperature of 55 °C and RH = 80%. For LoD estimation, the datapoints were fitted using a logarithmic function y(x) = a*log(x) + b.*

**Supplementary Section 17: Error bar calculation**

Error bars in **Figures 2e, 3c, S4, S7, and S9** represent a combined sample standard deviation triple calculated from 2 components:

*1st component:* Signal noise in $\Delta\lambda_{peak}$ calculated from 60 datapoints (3 min) taken before the 1st exposures to $H_2$ and 15 datapoints (45 s) from the end of the $H_2$ exposure pulses. The $\lambda_{peak}$ either in zero or non-zero $H_2$ concentration has a normal distribution ($\sigma_1$, $\sigma_2$). As a result, $\Delta\lambda_{peak}$ has a normal distribution as well with the variance $\sigma^2$ equal to $\sigma_1^2 + \sigma_2^2$.

*2nd component:* Repetition error estimated from the response differences for the same $H_2$ concentrations within each humidity level subset.

After that, these two components are combined using the standard approach, i.e., adding variances and taking the square root. In the end, the result is multiplied by the factor of three.

**Supplementary Section 18:** STEM images with EDS elemental linescans

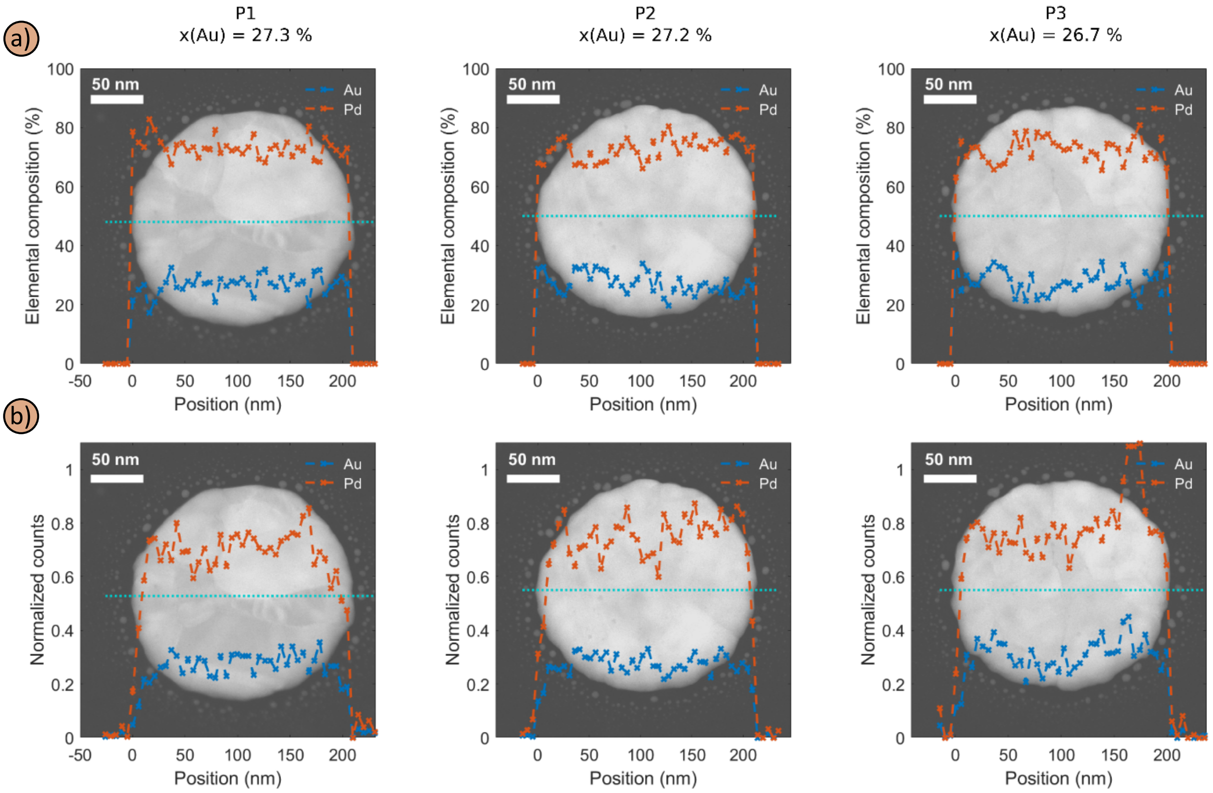


***Supplementary Figure 17.*** *STEM images of 3 randomly selected $Pd_{70}Au_{30}$ plotted together with EDS elemental linescans; a) elemental composition along the corresponding cyan dashed lines; b) integrated count areas under the Au(L) and Pd(L) EDS peaks normalized to count area sums at nanodisk centers.*

For each position along the profile, X-rays were collected for 5 s with 5 nm spatial distance in between points. The data was analyzed using FEI TIA. After background correction, the integrated peak intensities were acquired through standardless quantification.

**Supplementary Section 19: Optical absorption and desorption isotherm of the $Pd_{70}Au_{30}$ sensor in vacuum**

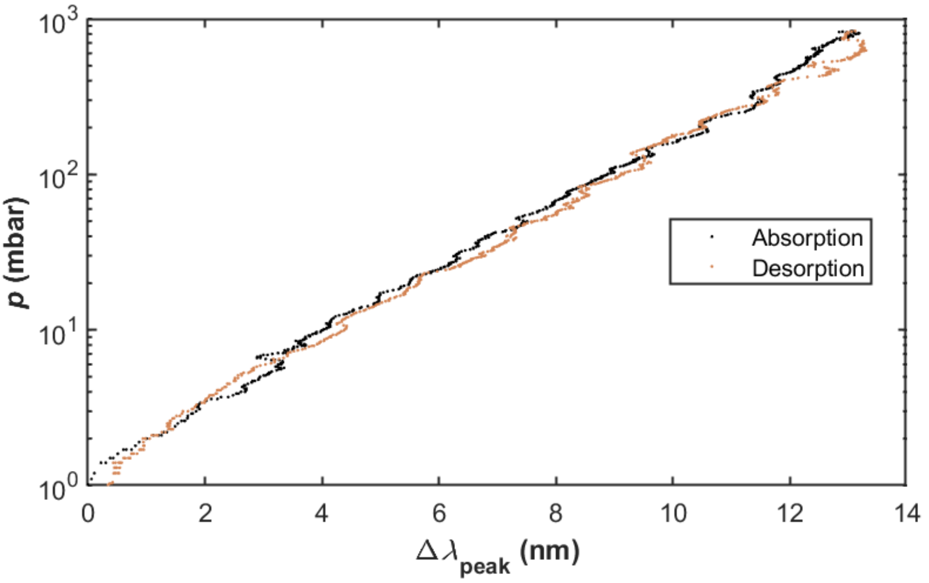


***Supplementary Figure 18.*** *Optical absorption and desorption isotherms measured in vacuum/pure hydrogen at absolute pressure and 30 °C for a typical $Pd_{70}Au_{30}$ sensor used in this work. It reveals the anticipated linear and hysteresis-free response of this alloy composition.*

**Supplementary Section 20: Repetitive self-referencing for drift correction in $\Delta\lambda_{peak}$ analysis**

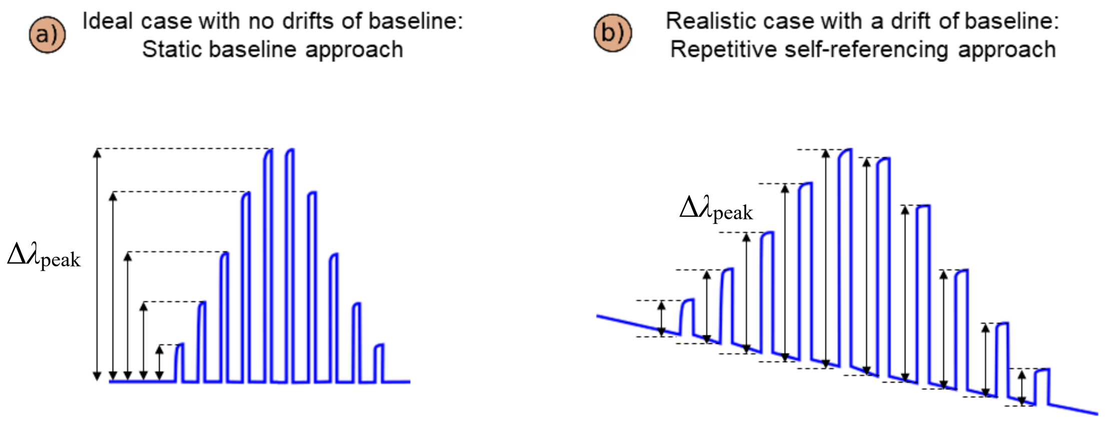


***Supplementary Figure 19.*** *a) Ideal sensor response without any drift over time and the possibility to use a static baseline approach; b) Repetitive self-referencing approach applied to the $\Delta\lambda_{peak}$ readout when the sensor baseline was exhibiting drift.*

## Supplementary Section 21: Long term stability test – regular pulse subsets in dry conditions

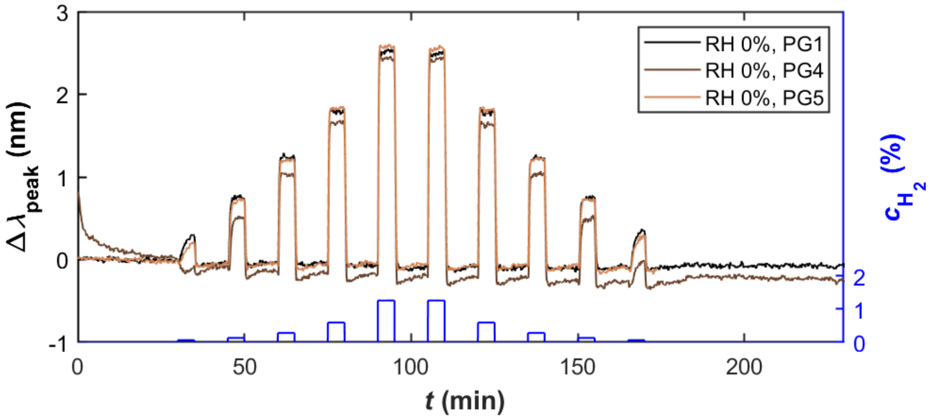


***Supplementary Figure 20.*** *Comparison of sensor responses at the beginning (Pulse Group 1, PG1) and at the end of the long-term stability test (PG4-5). Please note that PG4 is located right after the humidity level reduction (RH 80 → 0%), whereas PG5 takes place after an additional heating/drying step at 100 °C (the pulse group labels PG1, 4, and 5 refer to the ones used in* ***Figure 6c*** *in the main text). The comparison shows first that an additional heating/drying step at mildly elevated temperature is necessary to fully dry the sensor. Second, it shows that the sensing performance is unaltered by a long-term exposure to RH 80% at 80 °C.*

## Supplementary Section 22: Long term stability test – regular pulse subsets in humid conditions

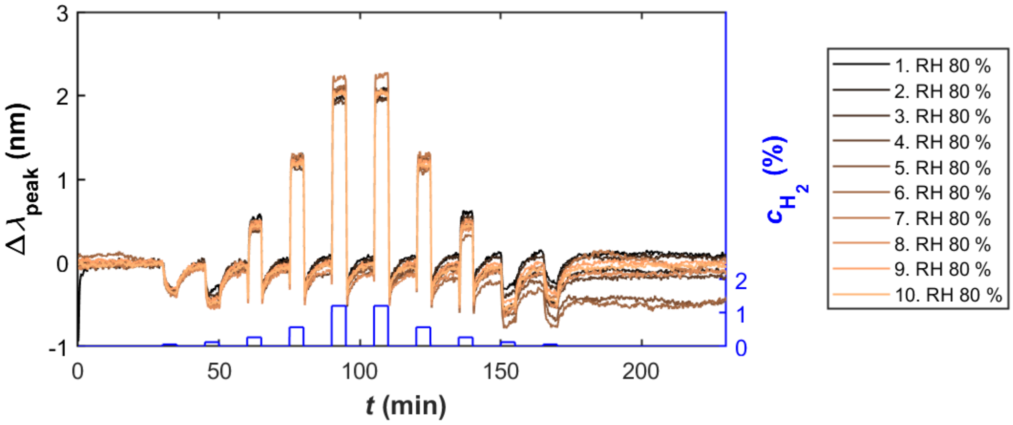


***Supplementary Figure 21.*** *Comparison of sensor responses to all regular $H_2$ pulse subsets at RH = 80% humid conditions in a chronological order. This figure zooms and overlays selected sections from* ***Figure 6b*** *in the main text. The comparison confirms sensor stability under the conditions of the experiment.*

**Supplementary Section 23: Transformer Architecture**

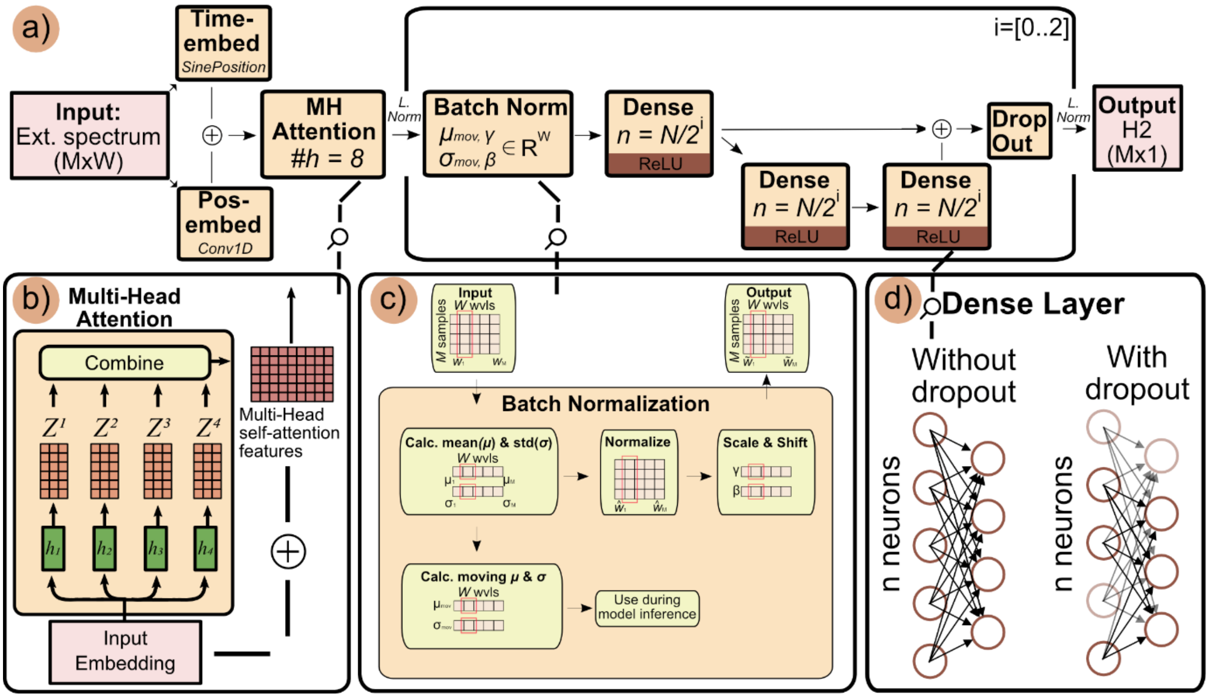


***Supplementary Figure 22.*** *Illustration of the Transformer-based architecture.*

Transformers, unlike feed-forward neural networks, are capable of recognizing patterns in data sequences and making predictions based on those patterns. They achieve this by encoding input data sequences and transforming the encoded information into meaningful outputs. The effectiveness of this architecture lies in its ability to handle sequential data by recognizing interdependencies between different data points. Here, each input sequence comprises a series of extinction spectra, where the transformer's self-attention mechanism allows it to weigh the importance of each extinction value of the spectrum in the sequence thus incorporating full spectral information for each mapping. Given the temporal nature of the data, this approach allows identifying strong correlations across different time steps, which would not have been possible with the simple 1:1 mapping of the DDNN approach we used first.

Since transformers operate on sequences rather than individual data points, they are generally less sensitive to drift and measurement noise. The time-series of extinction spectra is encoded through positional and time embeddings through a 1D-convolution and sinusoidal encoding, respectively (**Supplementary Figure 22**). These encodings feed into a multi-head attention module, where the different representations learned by the heads are then combined with the input embeddings and sent through a DDNN computational base as before, which is connected to an L1 norm loss and trained through backpropagation. Everything is implemented with TensorFlow.

**Supplementary Section 24: Transformer Training**

The Transformer network is trained, validated, and tested using the long-term stability measurement, split into bipartition sets of hydrogen pulses as for the DDNN case. The

model is trained and validated on 80% and 20% of a random permutation of datapoints in the first half of sequences of ramps of random hydrogen concentration, respectively. The test data consists of the second half of the corresponding sequences.

The Transformer network is trained using holdout validation with a patience of 150 epochs using the ADAM optimizer with an exponentially decaying learning rate from 1e-5 to 1e-6 with default parameters on a NVIDIA GeForce RTX 3080 GPU. The input during training is a randomly shuffled sequence of spectra and corresponding hydrogen concentrations in time with batch size 8192, wherein each spectrum-concentration pair is processed as a sequence rather than individual data points. This allows the Transformer to learn the temporal dependencies between different spectra-concentration pairs.

**Supplementary References**


(1) Ioffe, S.; Szegedy, C. Batch Normalization: Accelerating Deep Network Training by Reducing Internal Covariate Shift. arXiv 2015. https://doi.org/10.48550/ARXIV.1502.03167.

(2) Srivastava, N.; Hinton, G.; Krizhevsky, A.; Sutskever, I.; Salakhutdinov, R. Dropout: A Simple Way to Prevent Neural Networks from Overfitting. *J. Mach. Learn. Res.* **2014**, *15* (56), 1929–1958.

(3) Abadi, M.; Agarwal, A.; Barham, P.; Brevdo, E.; Chen, Z.; Citro, C.; Corrado, G. S.; Davis, A.; Dean, J.; Devin, M.; Ghemawat, S.; Goodfellow, I.; Harp, A.; Irving, G.; Isard, M.; Jia, Y.; Jozefowicz, R.; Kaiser, L.; Kudlur, M.; Levenberg, J.; Mané, D.; Monga, R.; Moore, S.; Murray, D.; Olah, C.; Schuster, M.; Shlens, J.; Steiner, B.; Sutskever, I.; Talwar, K.; Tucker, P.; Vanhoucke, V.; Vasudevan, V.; Viégas, F.; Vinyals, O.; Warden, P.; Wattenberg, M.; Wicke, M.; Yu, Y.; Zheng, X. {TensorFlow}: Large-Scale Machine Learning on Heterogeneous Systems. 2015. https://www.tensorflow.org/.

(4) He, K.; Zhang, X.; Ren, S.; Sun, J. Deep Residual Learning for Image Recognition. arXiv 2015. https://doi.org/10.48550/ARXIV.1512.03385.
